# Editorial: Advances in molecular diagnostics of allergy: novel allergens, biomarkers, and technical developments

**DOI:** 10.3389/fimmu.2026.1989471

**Published:** 2026-09-28

**Authors:** Youcef Shahali, Christiane Hilger, Martin D Chapman, Joana Vitte

**Affiliations:** 1 University Hospital of Besançon, Besançon, France; 2 Department of Infection and Immunity, Luxembourg Institute of Health, Esch-Sur-Alzette, Luxembourg; 3 InBio, Charlottesville, VA, United States; 4 University of Reims Champagne-Ardenne, INSERM UMR-S 1250 P3CELL, University Hospital of Reims, Immunology Laboratory, Reims, France

**Keywords:** allergen components, allergen immunotherapy, basophil activation test, biomarkers, immune monitoring, molecular allergology, multi-omics, precision allergy diagnostics

The rapid evolution of molecular allergology has transformed the diagnosis and monitoring of allergic diseases, moving beyond crude allergen extracts toward precise identification of individual allergen molecules, refined biomarkers of immune modulation, and advanced technical platforms. The first volume of this Research Topic brings together ten contributions that collectively advance our understanding of novel allergens, diagnostic biomarkers, and methodological innovations spanning food allergy, aeroallergens, immunotherapy monitoring, and emerging clinical entities. These works highlight both the promise and the remaining challenges in translating molecular insights into reliable clinical tools.

A central theme is the refinement of laboratory assays for monitoring allergen-specific immunotherapy (AIT) and oral immunotherapy (OIT). One study reports the development and pilot validation of a chemiluminescence immunoassay on the automated Autolumo A2000 Plus platform for simultaneous quantification of peanut-specific IgE and IgG4, including Ara h 2 and Ara h 6 (Yang et al.). The assay demonstrated high analytical sensitivity, precision, and strong concordance with the reference method ImmunoCAP, while capturing the expected decline in the sIgE/sIgG4 ratio during peanut OIT dose escalation. These findings support the potential utility of this platform for monitoring the sIgE/sIgG4 ratio as a dynamic marker of immune modulation, although larger prospective studies are needed to confirm its clinical predictive value. Complementary insights come from a comprehensive review of biomarkers for assessing AIT efficacy (Huang and Zeng). The authors compile evidence on immunoglobulins (sIgE, sIgG4, and their ratios), cellular readouts, functional cytokine assays, and provocation tests, advocating for composite scoring systems that integrate multiple parameters to predict early response and guide therapy. Together, these contributions underscore the shift from sensitization testing toward longitudinal immune monitoring.

Technical developments in allergen characterization and recombinant production are equally prominent. Transcriptomic and proteomic approaches were used to expand the pollen allergomes of four major grass species (*Anthoxanthum odoratum*, *Dactylis glomerata*, *Lolium perenne*, and *Poa pratensis*), identifying homologous counterparts to *Phleum pratense* allergens (groups 1–7 and 11–13), which were subsequently cloned and expressed in *E. coli* (Batard et al.). The enriched allergomic database substantially improved molecular resolution in the characterization of commercial extracts used for skin-prick testing, illustrating the practical value for quality control of *in vivo* diagnostic reagents. Another work examined the impact of expression host on recombinant Ara h 2 (Wangorsch et al.). Production in the yeast *Komagataella phaffii* introduced Kex2-mediated cleavage at a specific arginine site, with reducing conditions leading to the generation of peptide fragments lacking from *E. coli*-derived material. Although overall IgE reactivity and basophil-activating capacity remained largely preserved, subtle epitope differences were detected with selected monoclonal antibodies. This work underscores the importance of thorough physico-chemical and immunologic characterization of recombinant allergens before diagnostic deployment.

Functional cellular assays were investigated in a pediatric cohort comparing basophil activation test (BAT) performance with allergen extracts versus purified molecular components (Chhing et al.). Across 213 extract/component pairs spanning cow’s milk, wheat, house-dust mite, peanut, and other sources, extracts generally outperformed individual components as a first-step diagnostic tool, yielding higher rates of true-positive results consistent with clinical allergy status. Discordant extract-positive/component-negative outcomes were common, and even concordant pairs showed modestly lower activation with components. These data caution against using single molecular reagents in BAT as a first-step diagnostic strategy. As with serum IgE testing, component-based BAT should follow detectable sensitization to corresponding extract. The article demonstrates the importance of BAT to extract, particularly for cow’s milk, while highlighting modulating factors such as total IgE levels and concurrent immunotherapy.

Novel allergens and under-recognized clinical entities are addressed in several contributions. A case report and literature review document the first reported instance of cumin anaphylaxis triggered by inhalation in a pediatric patient, emphasizing the rising clinical relevance of spice allergy in the context of globalized food industry practices (Pessina et al.). Prick-to-prick testing with native spice powders proved diagnostically useful, although irritant effects and lack of standardized extracts remain limitations. The galactose-α-1,3-galactose (α-Gal) syndrome, a potentially severe IgE-mediated mammalian meat allergy associated with tick bites, is reviewed through its molecular and immunological pathways (Petry et al.). The authors integrate human, animal, and *in vitro* data to dissect how cutaneous exposure to α-Gal in tick saliva, as opposed to lifelong exposure to the same carbohydrate present in gut microbiota, drives Th2-biased class switching to α-Gal-specific IgE. Gaps in understanding the precise origin of α-Gal within ticks and the innate–adaptive immune interplay that favors sensitization are highlighted as priorities for biomarker discovery and prevention strategies.

Beyond traditional IgE-centric diagnostics, multi-omics and systems approaches are explored. Serum metabolomic profiling of carboxyl-containing metabolites in children with allergic rhinitis, asthma, or combined allergic rhinitis and asthma syndrome (CARAS) sensitized to house dust mites revealed both shared metabolic disturbances (potentially linked to immune dysregulation and gut dysbiosis) and disease-specific signatures: localized amino-acid perturbations in allergic rhinitis, systemic lipid remodeling in asthma, and a hybrid phenotype in CARAS (Zhang et al.). These findings suggest metabolomics may eventually aid differential diagnosis and endotyping of airway allergic diseases. Novel foods constitute a challenge for regulatory bodies to ensure consumer safety. Currently available tools to assess novel-food allergenicity being limited, advanced immune endotyping platforms are proposed, based on allergen-stimulated human cells (Assugeni et al.). Multi-parametric readouts including basophil activation (CD63), T-cell subsets (IL-5/IFN-γ ratios, CD154^+^ memory and regulatory populations), and complex signatures involving Th2 cells, memory Tregs, and homing markers, offer a path to distinguish true allergy from asymptomatic sensitization or healthy states, with potential translation to gut-on-a-chip models for safety evaluation of novel foods.

Finally, a bibliometric analysis of two decades of research on allergic diseases and B cells maps the intellectual landscape of this field (Mai et al.). Publication and citation trends show sustained growth, with keyword evolution from descriptive IgE associations toward tissue-associated B-cell biology and regulatory B cells. The analysis identifies growing translational interest in B-cell-targeted interventions and calls for standardized phenotyping in multicenter studies to support precision medicine.

Collectively, the articles in this Research Topic illustrate the multifaceted progress in molecular allergy and diagnostics. Significant challenges remain the standardization of biomarkers, validation of predictive models, and translation of complex immune signatures into routine clinical workflows. Continued interdisciplinary collaboration will be essential to convert these advances into more patient-centered diagnostic and monitoring strategies improving outcomes for individuals with allergic disease.

We thank all authors and reviewers for their contributions to this Research Topic and hope that the assembled work will stimulate further innovation in the field.

